# Time to first birth and its predictors among reproductive age women in high fertility countries in Sub-Saharan Africa: Inverse Weibull gamma shared frailty model

**DOI:** 10.1186/s12884-022-05206-9

**Published:** 2022-11-16

**Authors:** Wubshet Debebe Negash, Desale Bihonegn Asmamaw

**Affiliations:** 1 Department of Health Systems and Policy, College of Medicine and Health Sciences, Institute of Public Health, University of Gondar, P.O.Box: 196, Gondar, Ethiopia; 2 Department of Reproductive Health, College of Medicine and Health Sciences, Institute of Public Health, University of Gondar, Gondar, Ethiopia

**Keywords:** Time to first birth, Predictors, Reproductive age women, High fertility countries

## Abstract

**Background:**

Early initiation of childbearing leads to an increase in total fertility rate and population growth. It has been linked with both maternal and child morbidity and mortality. However, there is limited information on the timing of the first birth and its predictors in the area so far. Therefore, determining the time to first birth and its predictors will help to design strategies to improve fertility rate, maternal and child survival.

**Methods:**

The survey used recent (2010 – 2018) Demographic and Health data; a stratified, two-stage cluster sampling technique was used to select the sample. Inverse Weibull gamma shared frailty model was used to model the data at 95% confidence interval. Adjusted hazard ratio (AHR) and median hazard ratio (MHR) were reported as effect size. Statistical significance was declared at *p* value < 0.05.

**Results:**

The overall median age at first birth was found to be 19 years (IQR: 16, 21 years). Rural residency (AHR = 1.02, 95%, CI 1.00,1.04), agricultural employee (AHR = 1.14, 95%, CI 1.13, 1.17), and nonagricultural employee (AHR = 1.06, 95%, CI 1.05, 1.08), marriage below 15 years (AHR = 5.47, 95%, CI 5.37, 5.57) and 15–17 years (AHR = 3.27, 95%, CI 3.22, 3.32), had sex below 15 years (AHR =  = 1.57, 95%, CI 1.54, 1.61) and 15–17 years (AHR = 1.38, 95%, CI 1.38, 1.43), women who had unmet need for contraceptive (AHR = 1.39, 95%, CI 1.37, 1.42), and met need (AHR = 1.32, 95%, CI 1.30, 1.35), high spousal age gap (AHR = 1.17, 95%, CI 1.15, 1.19), not heard family planning message (AHR = 1.02, 95%, CI 1.01,1.04) were the higher hazard of early childbirth.

**Conclusion:**

The median age at first birth was found to be 19 years. This is lower than the optimal age for giving first birth, which is between late 20 s and early 30 s years. Rural residences, occupation, hearing family planning massage in the media, early sexual intercourse, early age at first marriage, high spousal gap, and unmet need for family planning were predictors of first birth at an early age. Thus, governments and non-governmental organizations should strive to implement programs that aim to reduce early age at first birth by considering these factors.

## Background

Age at first birth refers to the age of the mother when she gave birth to her first child and is a transition mark for women into motherhood [1]. It plays a significant role in the future life of each woman and has a direct relationship with fertility [2]. A woman’s age at which she begins childbearing can affect the number of children she will have, which in turn impacts the size, composition, and future growth of the population [3, 4].

Women who had their first child at a young age were more likely to have more children than those who had their first child later in life [5]. South Asia and sub-Saharan Africa (SSA) continue to have the highest proportions of child brides (44 percent and 18 percent, respectively) [6]. At age 18, 20% of women around the world, give birth to a child [7]. In developing countries, 2 million of the 7.3 million births to adolescents under the age of 18 are to girls under the age of 15 [8]. Whereas East Asia and the Pacific had a median birth age of 20.2 years [9]. Studies from the perspective of individual countries reveal different mean times of motherhood. For example, the median age at first birth in Ethiopia [10] and Nigeria [11] was 20 years. In Angola, 1 in 20 women age 15–19 had their first birth before age 15 [12].

Early childbearing affects the health of the mothers and their infants negatively [13–15], and may also have an economic impact on the family [3, 4]. It has consequences such as; poor prenatal health care, low birth weights, and higher mortality [16, 17]. The growing body of literatures revealed that the timing of first birth has both demographic and non-demographic effects on the woman throughout her lifetime [18]. First births before the age of 20 affect future health and increase all causes of maternal mortality [19, 20]. Research from 36 countries revealed the average relative risk of death in children under five years old is about 46% higher than in children born to mothers under 18, and 12% higher than in children born to mothers between 18 and 19, compared to children of mothers between 20 and 34 [21].

In various literatures, socio-demographic and economic factors were identified as predictors of age at first childbearing. These include early age at first sex [10, 22, 23], high spousal age differences [10], no formal education or lower levels of education [22, 24]. Early age at first marriage is one of the most consistent findings across the studies as predictors for early age at first birth [16, 25].

Determining the early age fertility benefits by providing comprehensive information about the timing of first birth and the reason behind early age delivery among reproductive age women in the high fertility SSA countries taking into account the correlated nature of the data. Therefore, the study will be useful to researchers and planners who wish to improve the health of mothers and children from a cluster effect perspective. Considering the above justifications, the main aim of this study was to investigate age at first motherhood and the predictors of early childbirth among reproductive-age women in high fertility Sub-Saharan Africa. This is further justified because early childbirth is the most important factor of population growth in sub-Saharan Africa [10, 26].

## Methods

### Study settings and data source

Community-based cross-sectional survey was conducted between January 2010 and December 2018 among reproductive age women in high fertility countries in SSA. Niger, Democratic Republic of Congo, Mali, Chad, Angola, Burundi, Nigeria, Gambia, and Burkina Faso were included in this study. These countries were selected because they are the top ten countries with high fertility rates in SSA, with fertility rates above 5.0, a value that is higher than the rate of 4.44 in Africa and 2.47 worldwide [27]. One country (Somalia) with no DHS data was excluded from the analysis.

After authorization was granted via an online request explaining the purpose of our study, we obtained data for these countries from the DHS program's official database, (https://dhsprogram.com). We used the woman’s record (IR file) data set and extracted the dependent and independent variables. The DHS is a nationally representative household survey that is conducted across low and middle-income countries every five years [28]. It has been an essential data source on issues of reproductive health in low and middle income countries as it gathers data on a number of reproductive health issues such as marriage, fertility, fertility preferences, and contraception [28].

Study participants were selected using a two-stage stratified sampling technique. Enumeration areas (EAs) were randomly selected in the first stage, while households were randomly selected in the second stage. Women declared infecund were excluded from this study.

### Study variables

The outcome variable of this study is the time to first birth (age in years) when a woman [15–49] years gave birth to her first childbearing until the data collection period [1, 2, 29]. The explanatory variables included socio-demographic and economic-related factors (educational status, employment status, residence, wealth index, and hearing family planning messages in the mass media), and age at first sex, age at first marriage, spousal age gap, and demand for contraceptives.

### Operational definitions

Event: giving first birth.

Censored: Not giving first birth.

Time to event/waiting time: it is the time taken in years (age) from her birth to age at first birth [1, 2, 10, 29].

### Data analysis

STATA version 14 Statistical software was used to extract, clean, code, and analyze data. Sample weights were done before further analysis, and descriptive statistics were described using frequencies, percentages, median, and interquartile range, and presented using tables, figures, and narratives. The Kaplan–Meier (K–M) method was used to estimate the time to first birth. The log rank test was used to compare survival experiences across categorical predictor variables and to reveal the statistical significance of the observed difference in the Kaplan–Meier survival plot. The Schoenfeld residual test was used to test the proportional hazard assumption.

Because the data were correlated at the cluster level, we used a shared frailty model to predict time to first birth among reproductive-age women in high fertility countries in SSA, assuming time to first birth is constant in the same clusters. Model adequacy was checked using Akaike Information Criteria (AIC).

Stratified analysis and a chi-square test were done for interaction terms. Finally, adjusted hazard ratio (AHR) was reported as a measure of effect size at 95% significant level and *p* value < 0.05. The median hazard ratio (MHR) was used to compare high and low risk clusters of time to early childbirth.

## Results

A total weighted sample of 186,771 reproductive age women were included in the study. The majority (22.23%) of the women were from Nigeria (Table 1).

**Table 1:** Description of Surveys and sample size characteristics in high fertility countries in SSA

Countries	Survey year	Weighted sample (n)	Weighted percentage (%)
Angola	2015/16	14357	7.69
Burkina Faso	2010	16978	9.09
Burundi	2016/17	17112	9.16
Chad	2014/15	17600	9.42
DR Congo	2013/14	37284	19.96
Gambia	2013	20348	10.89
Mali	2018	10465	5.60
Nigeria	2012	41525	22.23
Niger	2012	11102	5.94

### Baseline socio-demographic and reproductive characteristics of the study participants

A total weighted sample of 186,771 women was included in this study. Of the study participants, one-fifth (21.33%) were aged below 20 years. Three-fourths (75.49%) of the participants were married. Regarding residence, the majority (70.85%) of them were rural dwellers. The majority (74.67%) of the study participants had an age gap of five and above years with their partners (Table 2).

**Table 2:** Socio-demographic and reproductive health-related factors among reproductive age women in high fertility countries in SSA (*n* = 186,771)

Variables	Categories	Weighted frequency	Weighted percentage
Age in years	< 20	39826	21.33
	20–29	68854	36.87
	≥ 30	78081	41.80
Current marital status	Married	140989	75.49
	Unmarried	45782	24.51
Residence	Urban	70578	37.79
	Rural	116193	62.21
Age at first sex in years	< 15	50831	27.23
	15–17	98154	52.58
	≥ 18	37676	20.19
Age at first marriage in years	< 15	26353	18.69
	15–17	50350	35.71
	≥ 18	64287	45.60
Spousal age gap in years	< 5	32419	25.33
	≥ 5	95569	74.67
Modern contraceptive use	Yes	26348	14.11
	No	160423	85.89
Demand for contraceptive	No demand	64001	34.27
	Unmet need	35719	19.12
	Meet need	87051	46.61

### Socio-economic and information related characteristics

The result revealed that 78,169 (41.85%) of the respondents had not completed at least primary education. The majority (85.89%) of the participants were not used modern contraception (Table 3).

**Table 3:** Socio economic and information related factors among reproductive age women high fertility countries in SSA (*n* = 186,771)

Variables	Categories	Weighted frequency	Weighted percentage
Educational status of the respondents	No formal education	78169	41.85
	Primary education	43264	23.16
	Secondary and above	65338	34.98
Educational status of the partner	No formal education	62561	33.50
	Primary education	23411	12.53
	Secondary and above	100799	53.97
Occupation of the respondent	No working	53239	28.50
	Agricultural employee	48580	26.01
	Nonagricultural employee	84952	45.48
Occupation of the partners	No working	4122	2.21
	Agricultural employee	61679	33.02
	Nonagricultural employee	120970	64.77
Wealth index	Poor	68873	36.88
	Middle	36003	19.28
	Rich	81895	43.85
Media exposure	Yes	118920	63.67
	No	67851	36.33
Hearing family planning massage on media	Yes	132333	70.85
	No	54438	29.15
Modern contraceptive use	Yes	26348	14.11
	No	160423	85.89
Demand for contraceptive	No demand	64001	34.27
	Unmet need	35719	19.12
	Meet need	87051	46.61

### Time to first birth among respondents

**Fig. 1:**
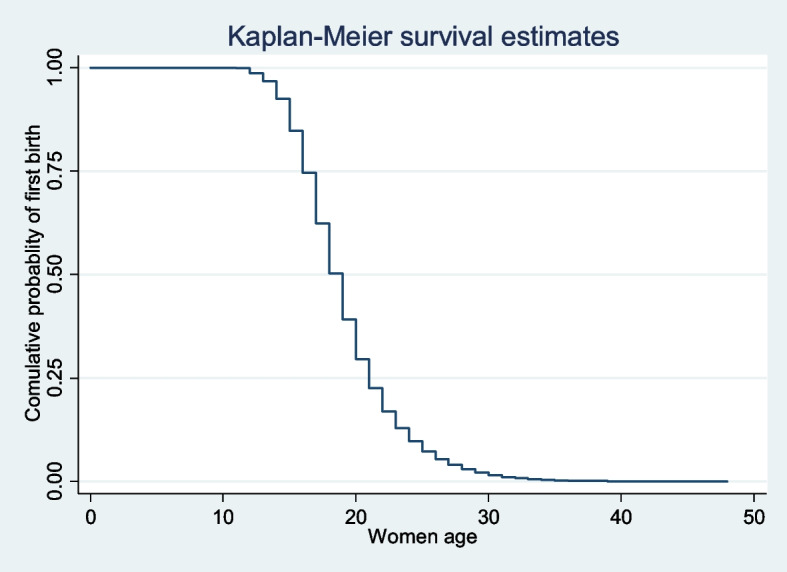
Kaplan–Meier failure estimates of time to first birth among reproductive-age women in high fertility countries in SSA

Majority (73.57%) of the study participants had given their first birth. The overall median time to their first birth was 19 years (IQR: 16, 21) (Fig. 1).

### Predictors of time to first birth among reproductive age women

Using the Kaplan–Meier failure function and the log rank test (X^2^), all predictors were determined at baseline. According to the log rank test, all the predictor variables showed significant survival differences at *p* = 0.001 (Table 4).

**Table 4:** Kaplan–Meier failure estimate and log rank test comparison of time to first birth among women in high fertility countries in SSA (*n* = 186,771)

Characteristics	Categories	N (%) weighted value	Ever given birth	Median (IQR) years	Log rank	*P*-value
Residence	Urban	70578	46387	19(17,22)	1472.71	0.001
	Rural	116193	91025	18(16,21)		
Age at first sex	< 15	50831	18696	16(15,18)	21539.05	0.001
	15–17	98154	87416	18(16,20)		
	≥ 18	37676	31243	21(20, 24)		
Age at first marriage	< 15	26353	25203	15(14,16)	77828.67	0.001
	15–17	50350	46879	17(16,18)		
	≥ 18	64287	59407	21(19,24)		
Spousal age gap	< 5	32419	30425	20(17,22)	2388.29	0.001
	≥ 5	95569	28908	18(16,21)		
Demand for contraceptive	No demand	64001	27518	18(16,22)	159.94	
	Unmet need	35719	32840	18(17,21)		
	Meet need	87051	77053	19(17,21)		
Occupation of the respondent	No working	53239	32316	18(16,21)	699.5	0.001
	Agricultural employee	48580	40676	19(17,21)		
	Nonagricultural employee	84952	64419	19(16,22)		
Hearing FP massage on media	Yes	132333	98545	18(16,21)	87.42	0.001
	No	54438	38866	19(16,21)		

### Possible model selection

#### Cox proportional hazard model

In bivariable analysis, seven predictors were significant at p-value of < 0.2 and then entered into the multivariable Cox model. Marriage was reduced from the model due to collinearity. After that, the Schoenfeld test for proportional hazard assumption was conducted. The proportional hazard assumption was violated in both the global test and log rank test due to the correlation of time to first birth. Due to this, Cox model was excluded. Secondly, the stratified Cox model was excluded because none of the predictor variables fulfilled the proportional hazard assumption in the model. In the end, the parametric models were included in this study (Table 5).

**Table 5:** Schoenfeld residual test for proportionality assumption of the Cox model among women in high fertility countries in SSA (*n* = 186,771)

Predictors	Rho	Chi^2^	Degree of freedom	Prob > chi2
Residence	0.01	24.02	1	< 0.001
Occupation of the respondent	0.00	4.04	1	0.04
Age at first sex	0.09	1048.05	1	< 0.01
Age at first marriage	0.027	6661.05	1	< 0.001
Demand for contraceptive	0.04	222.8	1	< 0.001
Spousal age gap	-0.01	23.21	1	< 0.001
Hearing family planning massage on media	0.01	21.13	1	< 0.001
**Global test**		**12924.96**	**7**	**< 0.001**

### Parametric survival model selection

#### Parametric shared frailty model

Variance of frailty (theta = 0) was statistically significant at *p* value of < 0.001, for all baseline hazard function with both inverse Gaussian and gamma shared frailty distribution. In other words, the frailty component influences the model and there is a correlation within the cluster. Finally, the inverse Weibull gamma shared frailty model was used for this study due to its lowest AIC (Table 6).

**Table 6:** Parametric shared frailty model comparison on time to first birth among reproductive age women in high fertility countries in SSA (n = 186,771)

Model	Log-likely hood	DF	AIC	Variance of theta	LR test of θ= 0
Inverse Weibull gamma	39,433.17	22	-78822.35	0.085	< 0.001^a^
Gompertz gamma	16,056.59	22	-32069.18	0.13	< 0.001

^a^ prefered model *AIC* Akakie information system, *DF* Degree of freedom

### Multi variable analysis of inverse Weibull gamma shared frailty model for time to first birth and its predictors

In the multivariable inverse Weibull gamma shared frailty model, there was a reduction of frailty from the null model (only with the cluster effect) of 0.19 to 0.084 in the full model (with predictor variables). Accordingly, residence, occupation of the respondent, hearing family planning massages in the media, age at first sex, age at first marriage, demand for contraceptive, and spousal age gap were significant predictors of age at 1^st^ birth at 95% confidence level.

Having the same level of frailty, women with agricultural employee and nonagricultural employee had 1.14 times (AHR = 1.14, 95%, CI 1.13, 1.17) and 1.06 times (AHR = 1.06, 95%, CI 1.05, 1.08) higher hazard of first birth at an early age as compared with no working women, respectively.

Women who had below 15 years at marriage and aged 15–17 years were 5.47 times (AHR = 5.47, 95%, CI 5.37, 5.57) and 3.27 times (AHR = 3.27, 95%, CI 3.22, 3.32) increases the hazard of first birth at an early age, respectively.

Women having first sex at the age of below 15 years increases the hazard of early childbirth by 1.57 times (AHR = 1.57, 95%, CI 1.54, 1.61) and 15–17 years 1.41 times (AHR = 1.41, 95%, CI 1.38, 1.43) than women aged 18 and above years keeping all other factors constant.

With the same level of frailty and keeping all other factors constant, women living in rural areas increase the hazard of early childbirth (AHR = 1.02, 95%, CI 1.00,1.04) than living in urban residents.

With the same level of frailty and adjusting for other factors, women who had an unmet need for contraceptives increases the hazard of early childbirth by 1. 39 times (AHR = 1.39, 95%, CI 1.37, 1.42), and met need by 1.32 times (AHR = 1.32, 95%, CI 1.30, 1.35) than no demand for contraception.

Women who had a spousal age gap five years and above had 1.17 times (AHR = 1.17, 95%, CI 1.15, 1.19) more hazard of early childbirth than their counterparts.

Given that on the same cluster and holding constant all other factors, women who have not heard family planning messages in the media had a higher hazard of early childbirth (AHR = 1.02, 95%, CI 1.01,1.04) (Table 7).

**Table 7:** Multivariable analysis of inverse Weibull gamma shared frailty model for time to first birth and among reproductive-age women in high fertility countries in SSA (*n* = 186,771)

Variables	Null model	First birth Status		Full model	
Log likelihood	12,178.38			39,215.03	
Effect size		**Event**	**Censored**	**CHR** ( 95%, CI)	**AHR** ( 95%, CI)
Residence					
Urban		46387	24191	1	1
Rural		91025	25168	1.22 (1.21, 1.24)	1.02 (1.00, 1.04)
Occupation of the respondent					
No working		32316	20923	1	1
agricultural employee		40676	7904	0.91 (0.89, 0.91)	1.14 (1.13, 1.17)
Nonagricultural employee		64419	20533	0.91 (0.89, 0.92)	1.06 (1.05, 1.08)
Age at first sex					
< 15		18696	32135	3.07 (3.01, 3.13)	1.57 (1.54, 1.61)
15–17		87416	10738	2.19 (2.16, 2.22)	1.41 (1.38, 1.43)
≥ 18		31243	6433	1	1
Age at first marriage					
< 15		25203	1150	6.24 (6.14, 6.33)	5.47 (5.37, 5.57)
15–17		46879	3471	3.67 (3.63, 3.72)	3.27 (3.22, 3.32)
≥ 18		59,407	4878	1	1
Demand for contraceptive					
No demand		27519	36483	1	1
Unmet need for contraceptive		32840	2879	1.20 (1.18, 1.22)	1.39 (1.37, 1.42)
Met need for contraceptive		77053	9998	1.15 (1.14, 1.17)	1.32 (1.30, 1.35)
Spousal age gap					
< 5		30425	1994	1	1
≥ 5		88908	6661	1.40 (1.38, 1.42)	1.17 (1.15, 1.19)
Hearing family planning massage on media					
Yes		98545	33788	1	1
No		38866	15571	0.92 (0.91, 0.94)	1.02 (1.01, 1.04)
Theta	0.19 (0.18, 0.21)				0.084 (0.075, 0.093)
MHR	1.52(1.503, 1.55)				1.32(1.30, 1.34)
LR test of theta = 0	< 0.001				< 0.001
Prob-hibar2	7602.79				3610.87

AHR Adjusted hazard ratio, CHR Crude hazard ratio, MHR median hazard ratio, LR Log rank

## Discussion

In the current study, the median age at first birth was found to be 19 (IQR = 16, 21) years in high fertility countries. The result of the study showed that residence, occupation of the respondent, age at first sex, age at first marriage, demand for contraceptives, spousal age gap, and hearing family planning messages in the media were identified as the predictive factors for time to first birth among reproductive age women in high fertility countries in SSA.

This finding is in line with results from Ethiopia (20 years) [10], Bangladesh (16.34 years) [30], Kenya (20.3 years) [31], Swaziland (18.22 years) [32], Nigeria (19 years) [11], and Uganda (19.2 years) [33]. This might be because, in these countries, early marriage and sexual intercourse activities at an early age are highly prevalent [10, 34, 35]. Early marriage compromises women’s reproductive health decisions, leading to early childbearing [36]. The other possible reason for this similarity might be the limited educational opportunities for girls in these countries since most of the population lives in rural areas, which forces them to get married at an early age, to get social and financial support [35, 37–39].

However, our finding was significantly lower than that of developed countries (> 30 years) [40, 41]. The possible explanation might be that adolescent girls in developed countries are more likely to stay in school for their adolescent age and a number of women go out to work for their economic independence, which helps mothers delay their first birth [42, 43]. Another possible reason could be that women in developed countries have good awareness about the consequences of early childbirth and have access to family planning to delay the first birth. Moreover, in developed countries, women have the right to exercise their reproductive rights and make their own reproductive health decisions [42–44]. Our result was also lower than the results from Egypt 22.6 years [45], and Ghana 21.4 years [46]. It may be due to differences in the prevalence of unmet need for family planning, the median age at first marriage and the age at first sexual intercourse [47, 48]. For example, unmet need for family planning in Egypt was 13% [45] whereas in high fertility countries in SSA, unmet need for family planning was 24.9% [48].

In this study, residence was one of the predictors for time to first birth. Women who lived in rural areas had higher hazards of having first birth at an early age than those who were lived in urban areas. This finding is similar to findings in Nigeria [11], Swaziland [32], Bangladesh [2], and Uganda [49]. The possible reason could be cultural malpractices like early marriage and abduction, which were highly prevalent in rural areas of SSA than their counterparts [37, 50]. Moreover, women in rural areas are less likely to be educated and less likely to be from educated parents, which means they have poor awareness of the consequences of early childbirth and a high unmet need for contraceptives [11, 37].

In this study, women’s occupation was inversely associated with early childbearing. This result was corroborated with study findings in the USA [51]. Possible explanations for the inverse association between occupational status and childbearing at an early age may be women who have their own work, are usually educated and have good awareness of family planning methods [52]. Furthermore, women who have their own jobs had the autonomy to make decisions related to household expenditure. This is important to understand as women who have access to spending money have improved reproductive decision making power and freedom of movement to access contraceptive services [53].

Hearing family planning massage in the media was associated with increased hazards of early age maternity compared to the counterparts. The reason for this could be that women who did not hear about family planning massage in the media might have had limited knowledge about contraception and the consequences of early childbirth [54].

Women who began sexual intercourse at an early age had higher hazards of having their first birth at an early age than those who began intercourse at a later age. This is in consistent with studies done in Ghana [55], Bangladesh [56] and Swaziland [57]. The possible explanation might be due to the exclusion of adolescents from education and sociocultural misconceptions regarding female sexual and reproductive health issues in these developing countries [39]. In addition, modern contraceptive use among early sexual initiators is lower than late initiators [38].

Age at marriage was also another predictor for age at first childbirth, as women got married early. The hazard of early childbearing at an early age was increased. This finding is in agreement with findings from Ethiopia [10, 34], Nigeria [11] and Bangladesh [2, 58]. This may be due to in developing countries, adolescent girl housewives are characterized by low educational attainment, low reproductive health knowledge, are economic dependent on their partners and less probability of autonomy in the decision-making process which fundamentally limit their ability to delay their childbearing to older ages [38, 59]. Moreover, early marriage increases the frequency of fertile sexual intercourses, and it leads to early childbearing [50, 60].

Regarding the spousal age, a higher spousal age gap significantly increased the hazard of early age at first birth compared to a low spousal age gap. This finding was coherent with the studies conducted in Ethiopia [10], Nigeria [61], and Bangladesh [62]. The possible explanation might be that a higher spousal age gap may lead to imbalanced power relations in the family and less probability of reproductive health discussions, including the decision to use family planning [53, 63].

Unmet need for family planning was found to be linked with higher early age maternity. This result was in agreement with reports in Nigeria [64], and Bangladesh [65]. The possible reason may be that sexually active women who have an unmet need for family planning may not be able to postpone unintended pregnancy and early births more often than those who do not have unmet need for family planning [34, 64].

The study's main strength was that it used nationally representative survey data and concentrated on high fertility countries in SSA. In addition, the DHS uses validated instruments in its appraisals of datasets along with its large sample size and well-designed procedures, such as training field enumerators and employing well-tested methods for data collection. However, DHS surveys are based on self-reported information and thus are prone to recall and social desirability bias. For example, there may be under-reporting of births that end in death. Furthermore, due to the use of secondary data, essential factors like socio-cultural factors were not available in the DHS data set. Hence, it was not possible to incorporate these variables.

Due to the high fertility rate, sub-Saharan Africa has contributed most of the world’s unexpected population dynamics. Strategies targeting early child birth plays a crucial role in helping to regulate population growth, and to improve the physical and economic wellbeing of women and their families as well as for the countries. However, in Sub-Saharan African countries with high fertility, 50% of reproductive age women give birth before their 19 years. Thus, thousands of reproductive age women died because of pregnancy related complications. Moreover, teenagers (10–19 years) are at higher risk for eclampsia, puerperal endometritis, and systemic infections, as well as low birth weight, preterm birth, and severe neonatal conditions. In order to combat the problem and to control total fertility rate, the respective country governments, nongovernmental organizations and policymakers should try to enhance access to contraception, particularly for women living in rural areas. Moreover, as a strategy for fertility reduction and maternal health improvement, women can delay first births by being empowered with job opportunities and regular family planning messages through mass media.

## Conclusion

In the current study, the median age at first birth was found to be 19 years, which is lower than the optimal age for giving first birth, which is between the late 20 s and early 30 s years [66–68]. Living in rural residences, early sexual intercourse, early age at first marriage, a high spousal age gap, and unmet need for family planning were predictors of first birth at an early age. On the other hand, occupation of the respondent and hearing family planning messages in the media were predictors of delayed first birth at an early age. Thus, governments and other responsible bodies should strive to implement programs to enhance access to contraception, particularly for women living in rural areas, to reduce unmet need for family planning. Since early childbirth, which often originated from early marriage, result in potential health risks for the young mother and their child, as well as blurred future job prospects. As a strategy for fertility reduction and maternal health improvement, women can delay first births by being empowered with job opportunity and regular family planning messages through mass media.

## Data Availability

Data for this study were sourced from Demographic and Health surveys (DHS), which is freely available online at (https://dhsprogram.com).

## Abbreviations

AIC: Akaike Information Criteria
AOR: Adjusted Odds Ratio
CI: Confidence Interval
DF: Degree of Freedom
DHS: Demographic and Health Survey
MHR: Median Hazard Ratio
SSA: Sub-Saharan Africa
WHO: World Health Organization
